# Quantifying gaps in the tuberculosis care cascade in Brazil: A mathematical model study using national program data

**DOI:** 10.1371/journal.pmed.1004361

**Published:** 2024-03-21

**Authors:** Sivaram Emani, Kleydson Alves, Layana Costa Alves, Daiane Alves da Silva, Patricia Bartholomay Oliveira, Marcia C. Castro, Ted Cohen, Rodrigo de Macedo Couto, Mauro Sanchez, Nicolas A. Menzies

**Affiliations:** 1 Harvard Medical School, Boston, Massachusetts, United States of America; 2 National Tuberculosis Programme, Ministry of Health, Brasilia, Brazil; 3 Health and Environment Surveillance Secretariat, Ministry of Health, Brasilia, Brazil; 4 Department of Global Health and Population, Harvard TH Chan School of Public Health, Boston Massachusetts, United States of America; 5 Department of Epidemiology of Microbial Diseases, Yale School of Public Health, New Haven, Connecticut, United States of America; 6 Department of Public Health, University of Brasilia, Brasilia, Brazil; 7 Center for Health Decision Science, Harvard TH Chan School of Public Health, Boston, Massachusetts, United States of America; PLOS Medicine Editorial Board, UNITED STATES

## Abstract

**Background:**

In Brazil, many individuals with tuberculosis (TB) do not receive appropriate care due to delayed or missed diagnosis, ineffective treatment regimens, or loss-to-follow-up. This study aimed to estimate the health losses and TB program costs attributable to each gap in the care cascade for TB disease in Brazil.

**Methods and findings:**

We constructed a Markov model simulating the TB care cascade and lifetime health outcomes (e.g., death, cure, postinfectious sequelae) for individuals developing TB disease in Brazil. We stratified the model by age, human immunodeficiency virus (HIV) status, drug resistance, state of residence, and disease severity, and developed a parallel model for individuals without TB that receive a false-positive TB diagnosis. Models were fit to data (adult and pediatric) from Brazil’s Notifiable Diseases Information System (SINAN) and Mortality Information System (SIM) for 2018. Using these models, we assessed current program performance and simulated hypothetical scenarios that eliminated specific gaps in the care cascade, in order to quantify incremental health losses and TB diagnosis and treatment costs along the care cascade. TB-attributable disability-adjusted life years (DALYs) were calculated by comparing changes in survival and nonfatal disability to a no-TB counterfactual scenario.

We estimated that 90.0% (95% uncertainty interval [UI]: 85.2 to 93.4) of individuals with TB disease initiated treatment and 10.0% (95% UI: 7.6 to 12.5) died with TB. The average number of TB-attributable DALYs per incident TB case varied across Brazil, ranging from 2.9 (95% UI: 2.3 to 3.6) DALYs in Acre to 4.0 (95% UI: 3.3 to 4.7) DALYs in Rio Grande do Sul (national average 3.5 [95% UI: 2.8 to 4.1]). Delayed diagnosis contributed the largest health losses along the care cascade, followed by post-TB sequelae and loss to follow up from TB treatment, with TB DALYs reduced by 71% (95% UI: 65 to 76), 41% (95% UI: 36 to 49), and 10% (95% UI: 7 to 16), respectively, when these factors were eliminated. Total health system costs were largely unaffected by improvements in the care cascade, with elimination of treatment failure reducing attributable costs by 3.1% (95% UI: 1.5 to 5.4). TB diagnosis and treatment of false-positive individuals accounted for 10.2% (95% UI: 3.9 to 21.7) of total programmatic costs but contributed minimally to health losses. Several assumptions were required to interpret programmatic data for the analysis, and we were unable to estimate the contribution of social factors to care cascade outcomes.

**Conclusions:**

In this study, we observed that delays to diagnosis, post-disease sequelae and treatment loss to follow-up were primary contributors to the TB burden of disease in Brazil. Reducing delays to diagnosis, improving healthcare after TB cure, and reducing treatment loss to follow-up should be prioritized to improve the burden of TB disease in Brazil.

## Abstract 2

Using a mathematical model, Sivaram Emani and colleagues quantify the gaps in the tuberculosis care cascade in Brazil.

## Author summary

**Why was this study done?:**

- In Brazil, many people with tuberculosis (TB) do not receive proper care due to delayed diagnosis, ineffective treatment, or loss to follow-up.
- Care cascade analysis can be used to understand the TB burden of disease, providing insights for policy generation and public health strategy.

**What did the researchers do and find?:**

- We used a care cascade model to estimate the health losses and TB program costs attributable to each gap in the care cascade for TB disease in Brazil.
- We found that delayed diagnosis, post-TB health sequelae, and loss to follow-up were the main contributors to TB disease burden.

**What do these findings mean?:**

- Our findings underscore the potential benefit of reducing delays in diagnosis, improving health outcomes after TB cure, and enhancing retention in TB treatment to alleviate the burden of TB disease in Brazil.
- This study did not consider social factors, although these are known to be important determinants of TB disease outcomes.

## Introduction

Tuberculosis (TB) remains one of Brazil’s largest infectious disease challenges with 104,000 individuals estimated to have developed TB in 2021 and 8,000 dying with the disease [1]. While TB incidence in Brazil has declined slowly over past decades, this decline has reversed since 2015, related to an economic recession that began in 2014 [2], and more recently due to the impacts of the Coronavirus Disease 2019 (COVID-19) pandemic [1]. The current TB incidence rate in Brazil (31 per 100,000) is approximately 50% higher than the regional average [1], though lower than most other large low and middle-income countries. Brazil is included as one of 30 high-TB burden countries identified by the WHO, reflecting the importance of Brazil achieving global TB elimination goals [3]. Within Brazil, the risks of developing TB vary systematically as a function of demographic, behavioral, and social factors, with TB concentrated within impoverished and marginalized populations [4,5]. There are also large differences in incidence rates across states and municipalities, likely related to differences in socioeconomic determinants as well as variation in the strength of TB case detection [2,6]. In Brazil, TB diagnosis and care is provided for free though the publicly funded Sistema Único de Saúde (SUS), with diagnosis and treatment for uncomplicated cases provided through primary care, and inpatient treatment for complicated cases. Guidelines for TB diagnosis and care are determined by the federal Ministry of Health, following WHO-recommended approaches. States are responsible for the delivery of health services, resulting in variation between states in how TB services are organized.

Providing prompt and effective treatment is a central pillar of TB control in Brazil [7], following the tenets of the Global End TB Strategy [8]. In 2021, TB treatment coverage (new and previously treated individuals initiating treatment as a percentage of total TB incidence) was estimated to be 76% [1]. Early diagnosis and treatment for individuals who develop TB disease is important for reducing morbidity and mortality during the disease episode and minimizing risks of ongoing disability for TB survivors [9]. Prompt initiation of treatment also reduces the duration of infectiousness and minimizes onward transmission. Even after treatment initiation, adherence to and completion of the treatment regimen are needed to achieve high cure rates and prevent the acquisition of drug resistance. Unfortunately, many individuals who develop TB do not receive appropriate care, either through delayed or missed diagnosis, use of an ineffective treatment regimen, poor adherence, or loss to follow-up before treatment completion [10]. Strengthening access to and completion of treatment is a major TB program priority for Brazil and other high-burden countries [3].

Identifying the most effective strategies to strengthen TB detection and treatment requires an understanding of the dynamics of patient care, from initial development of disease to cure or death. The TB care cascade is a conceptual framework that describes the steps required to provide effective TB diagnosis and treatment, and the losses that can occur at each step of this process [11,12]. Moreover, this framework can be used to represent outcomes for individuals at higher risk of adverse outcomes (individuals with risk factors for rapid disease progression, individuals with TB drug resistance), as well as individuals without TB disease who may be mistakenly enrolled in care through false-positive diagnosis [13]. Care cascade models have been used in several prior studies, including descriptive analyses of programmatic performance in India [14] and South Africa [15], and counterfactual analyses of possible care cascade improvements in India, Kenya, and Moldova [16].

In this study, we used a care cascade model to analyze the dynamics of TB disease and treatment in the Brazilian health system, parameterized with data on TB service coverage and outcomes for 2018. Using this model, we quantified how TB treatment outcomes—and the health losses attributable to TB—vary across Brazil according to age, sex, state, human immunodeficiency virus (HIV) status, and drug resistance status. We also report how health losses and costs can be attributed to each step in the TB care cascade to guide decision-making about where programmatic improvements can be focused to maximize the effectiveness of TB services.

## Methods

### Study population

The study population included 2 distinct groups: (i) individuals with incident TB disease (“TB cohort”), for whom appropriate care is characterized by prompt diagnosis and successful TB treatment; and (ii) individuals with non-TB health conditions (e.g., bacterial pneumonia, lung cancer) who present with TB-like symptoms and are evaluated for TB disease (“non-TB cohort”), for whom appropriate care is characterized by prompt diagnosis and treatment of their non-TB health conditions [13]. We included both groups in the analysis as together they represent the population potentially affected by changes in Brazil’s TB treatment program. For this study, we focused on data for 2018, so that the outcomes of TB treatment (e.g., death, cure) were reliably represented in national databases.

### Data

Data were derived from Brazil’s Notifiable Diseases Information System (SINAN) [17] and the national Mortality Information System (SIM) [18]. The SINAN database contains information on all individuals diagnosed with TB in Brazil, including demographics and TB risk factors, diagnostic testing results, HIV coinfection testing, and treatment outcomes (e.g., loss to follow-up, treatment failure, cure, death, misdiagnosis). Each case is also classified based upon whether the individual has had TB previously, is returning to care after being lost to follow-up, or has not previously been diagnosed with TB. Each observation in SIM represents a death reported by death certificate at the municipal level. SIM records demographic information as well as cause of death, representing a near-complete record of deaths in the country [19]. We extracted data on TB case notifications reported in SINAN and mortality data for individuals dying with TB (including deaths with comorbid conditions) from SIM for the year 2018 and used these to represent total TB diagnoses and deaths for the cohort developing TB in 2018. As deaths due to incident TB will be spread over current and future years, this approach could be biased if the number of TB deaths were changing over time. To validate our approach, we checked the number of SIM TB deaths in the following year (2019), finding the number of SIM deaths to be stable over the period of interest (SIM TB deaths in 2019 within 2% of those reported for 2018).

### Mathematical model

**Fig 1:**
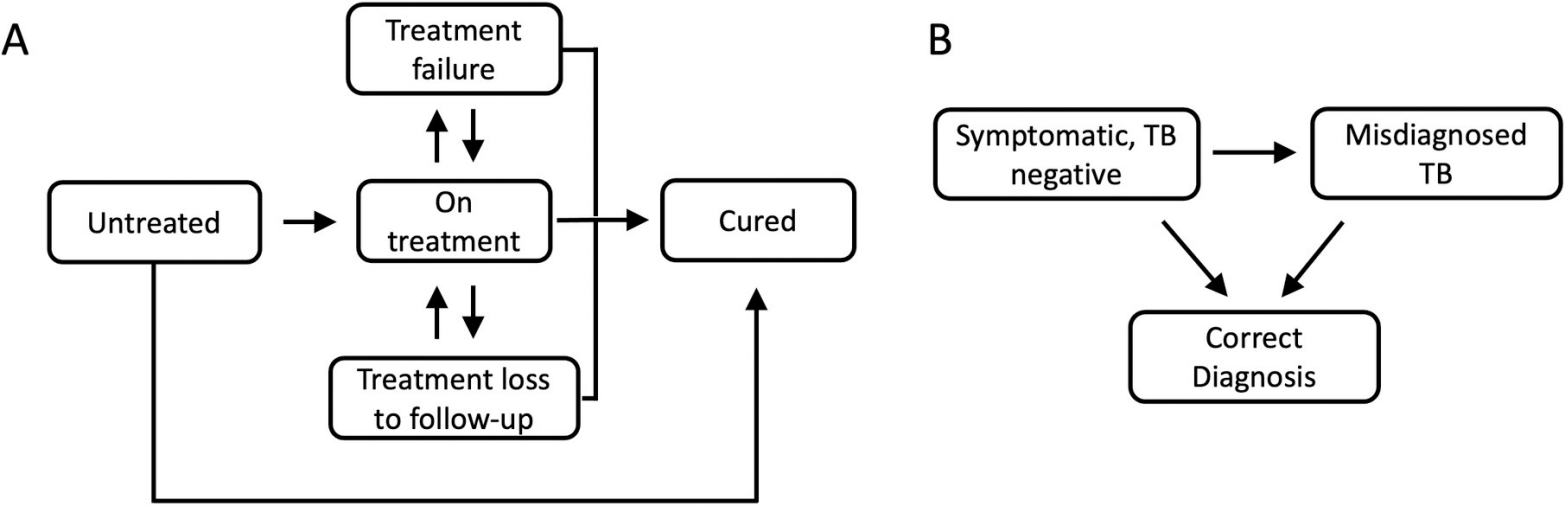
Simplified schematic of care cascade for TB (A) and non-TB (B) cohorts. Transitions to the death state are omitted and implied from every depicted health state in the TB cohort. TB, tuberculosis.

We developed 2 Markov state transition models representing the TB cohort and non-TB cohort, described above [20]. In both models, the starting cohort transitions through discrete health states over time to reproduce observed patterns of TB natural history and healthcare utilization. Health states and transitions for each model are shown in Fig 1 (additional detail shown in S1 Fig). Key parameters determining model transitions are given in S1 Table. Models were simulated with a one-month timestep.

In the TB cohort, individuals begin in the “untreated” state, where they can die with background mortality, self-cure (i.e., control the disease without treatment), or present for TB diagnosis. Individuals transition to the “on treatment” state following presentation for TB diagnosis, true-positive diagnosis, and successful treatment initiation (determined by the state-level primary loss to follow-up probability). Sensitivity and specificity of the TB diagnostic algorithm was derived based upon SINAN reporting of utilization of diagnostic tests (bacteriological, clinical/radiographic) as well as literature on performance of these testing modalities. Individuals initiating treatment with rifampicin-susceptible TB are assumed to receive a standard 6-month first-line treatment regimen, and can either complete the regimen, be lost to follow-up before treatment completion, acquire drug resistance, or die during treatment. Patients in the “treatment loss to follow-up” state are assumed to have active disease and can die, self-cure, or reinitiate treatment. Patients completing treatment can transition to the “cured” state, reinitiate treatment following identified treatment failure, or transition to the “unidentified treatment failure” state with ongoing active disease. Individuals in the unidentified treatment failure state can either die, self-cure, or reinitiate treatment.

The model is stratified by 5-year age group (0–4, 5–9, … 85+), HIV status (HIV coinfected, HIV uninfected), TB drug resistance status (rifampicin-susceptible, rifampicin-resistant), and state of residence (26 states + federal district). HIV status and state of residence are assumed fixed over the simulation period. Background and TB-specific mortality rates are assumed to vary with age and HIV status. HIV-associated mortality was modeled as a rate ratio applied to underlying mortality. This rate ratio varied between states based on the fraction of HIV–positive individuals diagnosed with TB in SINAN that were receiving antiretroviral therapy (ART) at the time of diagnosis. A fraction of the incident TB cohort is assumed to have rifampicin-resistant TB (RR-TB). Upon TB diagnosis, a fraction of individuals with RR-TB are assumed to receive drug sensitivity testing and be initiated on an appropriate (second-line) treatment regimen. The remainder is initiated on an inappropriate (first-line) treatment regimen. A fraction of individuals on a first-line regimen who are identified as having failed treatment are tested for drug resistance and initiate second-line treatment, with the rest of identified treatment failures continuing first-line therapy. Second-line regimens are assumed to be on-average 18 months [21].

The TB cohort model is additionally stratified by early/late TB disease to model increasing disease severity. Individuals with incident TB disease begin in the “early TB” stratum, which is characterized by asymptomatic disease (only exposed to background mortality), and transition to the “late TB” stratum where mortality is compounded by disease-specific mortality. The progression rate from early to late disease was assumed to be higher for individuals with HIV and assumed to be zero while individuals were receiving TB treatment. Individuals with late TB also experience elevated mortality rates after TB cure (representing long-term health consequences precipitated by the disease episode) [9,22]. Individuals with late TB also have higher rates of care-seeking and lower rates of self-cure.

Individuals in the “non-TB” cohort either receive a true-negative diagnosis and transition to the “correct diagnosis” state or a false-positive diagnosis and transition to the “TB treatment” state. Those receiving treatment are assumed to receive a 6-month treatment regimen and experience increased mortality due to their untreated underlying condition and possible TB treatment side effects. They exit treatment with the same rates as individuals with TB disease (including treatment completion and loss to follow-up) and remain in the off-treatment state for the rest of the simulation.

### Model calibration

We calibrated the TB cohort model to local TB services and disease outcomes data: (i) the number of patients initiating TB treatment by age and state (SINAN); (ii) the number of deaths with TB during TB treatment by age (SINAN); (iii) total TB deaths by state (SIM); and (iv) HIV-coinfected TB treatment deaths (SINAN). SINAN-reported deaths and loss to follow-up notifications were adjusted according to a previous linkage study, which show overreporting of mortality in SINAN, likely due to misreporting of treatment loss to follow-up as death [23]. These targets were used to calibrate parameters determining TB incidence by age, state, and HIV status, TB mortality rates, rate of presentation for diagnosis for individuals with untreated TB by state, and the mortality rate ratio of HIV-coinfected versus HIV-uninfected TB. The rate of presentation was calibrated as a regression with the ratio of individuals treated to dead with TB (treatment-death ratio) by state. The number of individuals in the non-TB cohort was calculated assuming a false-positive rate among SINAN treatment notifications derived from expert consensus and from the sensitivity, specificity, and prevalence of TB among those presenting with TB-like symptoms [13]. We fit the model using a Bayesian calibration approach [24]. Using a Nelder–Mead optimization routine, we minimized an objective function that considered the sum of squared deviation between modeled outcomes and calibration targets as well as probability densities for the model parameters.

S2 Fig shows the calibrated mortality rates for individuals in different disease states. S2 Table shows the values for rate of presentation for diagnosis, loss to follow-up rate, and primary loss to follow-up fraction by state, obtained using both calibration and direct derivation from programmatic data. S3 Fig depicts model fit to empirical data on TB services and outcomes: deaths on TB treatment by age and HIV, and total TB deaths by state.

In addition, we validated model estimates of case fatality at 1 year after TB treatment initiation against a linked SINAN-SIM dataset that was available for 2015. To do so, we simulated a cohort of individuals from the point of treatment initiation for 12 months and compared the modeled case fatality estimates against empirical case fatality estimates from the linked SINAN-SIM dataset by age group and HIV status. S4 Fig shows the results of this validation.

### Model outcomes

We estimated the following outcomes: TB incidence, patients initiating treatment, patients lost to follow-up, treatment failures, deaths with TB, self-cures, years of life lost (YLLs), years lived with disability (YLDs), disability-adjusted life years (DALYs, the sum of YLLs and YLDs), and programmatic costs (2021 USD) of diagnosis and treatment. Disability weights and costs used for calculation of model outcomes are shown in S1 Table. Outcomes were stratified by age at acquisition (incidence) of active TB disease (5-year age group), time since disease incidence, state of residence, drug resistance status, and HIV status. In addition, we report the total time spent with infectious TB disease as an indicator of the potential for onward transmission. This was calculated as the sum of time spent in all untreated TB disease states, given that infectiousness is high even in subclinical (early) disease and declines rapidly with the initiation of treatment [25–27].

### Analytic scenarios

The calibrated model described above was used as a baseline scenario to simulate lifetime outcomes of the study cohort under current program performance. We simulated several alternative scenarios to compare to this baseline. Each of these scenarios represented improvements at a single step in the TB care cascade to ideal program performance (e.g., diagnostic sensitivity set to 100% or no treatment loss to follow-up). We calculated incremental differences in cohort outcomes between these scenarios and the baseline to quantify changes in health and cost outcomes at each step of the TB care cascade. As described above, TB-attributable DALY calculations scenarios are performed by comparing to a control scenario with no TB-associated mortality or morbidity. S3 Table provides additional details for each scenario.

### Statistical analysis

We specified probability distributions representing the uncertainty associated with each model parameter and propagated the uncertainty in these inputs using a second-order Monte Carlo simulation [28]. Parameters defined in [0, 1] (e.g., probabilities) were represented with beta distributions while parameters defined in [0, ∞] (e.g., rates) were represented with gamma distributions. Uncertainties quantified in this analysis include parametric uncertainty related to parameter distributions, defined based on published values or expert opinion (S1 Table). Uncertainties not quantified in our analysis include those inherent to model structure (e.g., fixed rate of treatment completion) and assumptions regarding data integrity (e.g., every positive diagnosis results in a SINAN treatment notification). Where measures of uncertainty were not available for a given parameter, we assumed an interval equivalent to +/− 50% of the point estimate. We drew a Latin hypercube sample (*n* = 1,000) from these probability distributions and used the model to simulate study outcomes for each parameter set [29]. This analysis generated 1,000 estimates for each study outcome. We calculated point estimates for each outcome as the mean of these 1,000 values and calculated equal-tailed 95% uncertainty intervals (UIs) as the 2.5th and 97.5th percentiles of these distributions. We also calculated partial rank correlation coefficients (PRCCs) to quantify the strength of each parameter’s association with major study outcomes, controlling for other parameters [30].

## Results

### Current program performance

Table 1 reports results for major study outcomes under current TB program performance. Total TB incidence was estimated as 73,900 (95% UI: 67,200 to 80,600) for 2018, of which 90.0% (95% UI: 85.2 to 93.4) initiated treatment. Incidence estimates by state and age group are provided in S5 Fig. Total deaths with TB were estimated as 7,402 (95% UI: 5,596 to 9,174), corresponding to a case fatality of 10.0% (95% UI: 7.6 to 12.5) for the overall cohort. Among the cohort with TB, the case fatality for individuals with comorbid HIV was 29.9% (95% UI: 20.9 to 36.8), and the case fatality for those with RR-TB was 25.1% (95% UI: 19.8 to 30.4). The case fatality for individuals with TB who never initiate TB treatment was 27.0% (95% UI: 16.4 to 41.2), with the remaining 73.0% (95% UI: 59.8% to 83.6%) of these individuals achieving self-cure. Of the total deaths with TB, 62.1% (95% UI: 56.2 to 66.6) occurred among individuals receiving TB treatment. Of all of individuals cured of TB, 90.5% (95% UI: 84.8 to 94.6) were cured via treatment (versus self-cure). The time spent by patients in various TB states is shown in S6 Fig.

**Table 1:** Estimated TB care cascade outcomes under current programmatic performance.

Parameter	Value (95% UI)
Total TB incidence	73,900 (67,200–80,600)
RR-TB incidence (%)	1.6 (1.0–2.2)
# TB negative with TB-like symptoms	89,900 (30,800–293,500)
# Initiating treatment	66,500 (61,000–71,500)
Percent initiating treatment	90.0 (85.2–93.4)
Percent initiating treatment—HIV+	87.7 (83.4–91.6)
Percent initiating treatment with late (vs. early) TB	73.8 (62.9–81.5)
Fraction lost to follow up before treatment	5.7 (4.3–7.4)
Percent of treatments lost to follow up	17.5 (14.0–21.1)
Percent of treatments lost to follow up—RR TB	25.7 (20.9–30.4)
# Deaths with TB	7,402 (5,596–9,174)
Case fatality rate (%)	10.0 (7.6–12.5)
Case fatality rate—HIV+ (%)	29.9 (20.9–36.8)
Case fatality rate—RR TB (%)	25.1 (19.8–30.4)
Percent of all TB deaths with HIV coinfection	27.0 (21.1–30.6)
Percent of all TB deaths with RR-TB	5.1 (3.7–7.0)
Percent of all TB deaths with late (vs. early) TB	95.7 (93.5–96.9)
# Deaths on treatment	4,403 (3,346–5,368)
Percent of total deaths on treatment	62.1 (56.2–66.6)
Treatment fatality rate (%)	5.3 (4.1–6.5)
Untreated TB fatality rate (%)	27.0 (16.4–41.2)
TB attributable DALYs (thousands)	255 (208–306)
TB attributable YLLs (thousands)	186 (145–229)
TB attributable YLDs (thousands)	69 (52–89)
TB DALYs per incident case	3.5 (2.8–4.1)
TB DALYs per incident HIV+ case	5.0 (4.1–5.8)
TB DALYs per incident RR TB case	7.1 (5.8–8.5)
DALYs per false-positive case	0.03 (0.02–0.03)
Percent of cures with late (vs. early) TB	71.6 (60.9–79.2)
Percent of cures via self-cure (vs. treatment)	9.5 (5.4–15.2)
Percent self-cure with late (vs. early) TB	20.8 (14.0–30.4)
Total programmatic cost—TB cohort (millions USD)	97 (75–122)
Total programmatic cost—non-TB cohort (millions USD)	11 (4–25)
Diagnostic cost per incident TB case (USD)	59 (23–110)
Treatment cost per incident TB case (USD)	1,250 (980–1,570)
Cost per incident RR-TB case (USD)	10,120 (7,450–13,100)
Cost per false-positive TB case (USD)	835 (636–1,038)
Average time with undiagnosed TB (mo)	6.0 (5.0–7.3)
Average duration of disease (mo)	13.3 (12.3–14.9)
Cumulative transmission burden (thousands person-years)	45 (38–57)

DALY, disability-adjusted life year; HIV, human immunodeficiency virus; RR, rifampin resistant; TB, tuberculosis; USD, United States dollars; YLD, years lived with disability; YLL, years of life lost.

Total TB-attributable DALYs—calculated by comparing the base-case scenario to a no-TB counterfactual—were estimated at 255,000 (95% UI: 208,000 to 306,000), corresponding to 3.5 (95% UI: 2.8 to 4.1) DALYs per incident TB case, 5.0 (95% UI: 4.1 to 5.8) DALYs per individual with TB-HIV coinfection, and 7.1 (95% UI: 5.8 to 8.5) DALYs per individual with RR-TB. Table 1 also reports results for individuals without TB who present with TB-like symptoms. The DALYs attributable to false-positive diagnosis per false-positive case were estimated to be 0.03 (95% UI: 0.02 to 0.03). Total time spent with infectious TB disease (an indicator of the potential for onward transmission) was 45,000 person-years (95% UI: 38,000 to 57,000).

**Fig 2:**
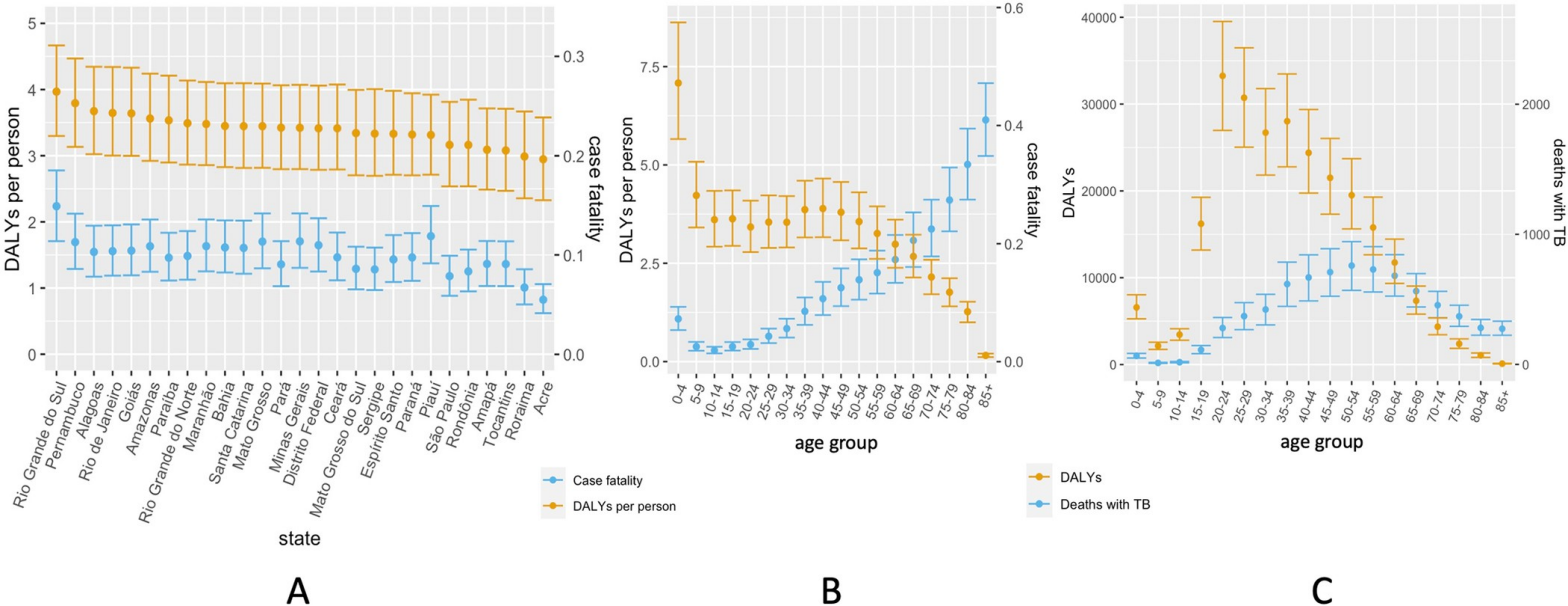
Per person burden of TB disease by age at TB disease incidence (A) and state (B), as measured by TB case fatality and TB attributable DALYs. Total deaths with TB and TB attributable DALYs by age at TB disease incidence (C). Point and whiskers indicate median and 95% UI, respectively, in simulations of parametric uncertainty. DALY, disability-adjusted life year; TB, tuberculosis; UI, uncertainty interval.

Fig 2 shows results for deaths with TB and lifetime DALYs attributable to TB, stratified by state and the age at TB disease incidence. Total DALYs due to TB were highest among adults acquiring TB disease at age 20 to 24 years, and TB deaths were highest among adults aged 50 to 54. The average DALYs per person were highest among infants, and while case fatality increased with age, case fatality was also estimated to be high for young children. Rio Grande do Sul was the state with highest case fatality (14.9% (95% UI: 11.4 to 18.5)) and DALYs per person (4.0 (95% UI: 3.3 to 4.7)), while the lowest values for these outcomes were estimated for Acre (case fatality 5.5% (95% UI: 4.1 to 7.1), DALYs per person 2.9 (95% UI: 2.3 to 3.6)).

**Fig 3:**
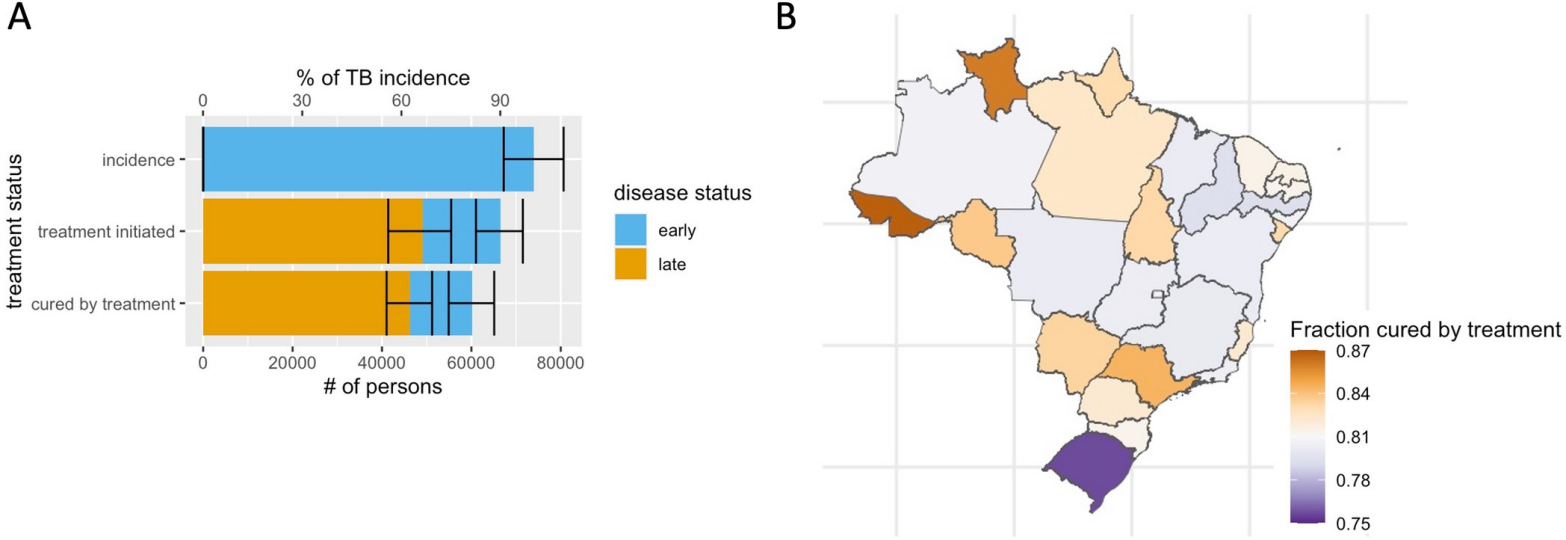
Fraction initiating treatment and cured by treatment with early vs. late disease. (A). Fraction cured by treatment by state of residence (B). Brazil state shapefiles obtained from geobr package (url: https://github.com/ipeaGIT/geobr). Error bars indicate median and 95% UI, respectively, in simulations of parametric uncertainty. TB, tuberculosis; UI, uncertainty interval.

Fig 3A shows the overall health system performance for achieving prompt (in early disease) treatment for individuals with TB. Of the 90.0% (95% UI: 85.2 to 91.6) initiating treatment, 26.2% (95% UI; 18.4 to 37.1) initiated treated with early TB, and of the 81.4% (95% UI: 76.3 to 85.6) cured by treatment, 23.1% (95% UI: 16.2 to 33.1) were cured with early TB. Fig 3B shows health system performance of the care cascade at the state level, as measured by the fraction of incident TB cases successfully completing the case cascade (accessing care, receiving a TB diagnosis, and achieving TB cure via treatment), which ranged from 75.7% in Rio Grande do Sul to 86.8% in Acre.

The estimated total programmatic costs of TB care are presented in Table 1. The programmatic cost for the TB cohort was estimated to be $97 million USD (95% UI: 75 to 122) of which $5 million (95% UI: 2 to 8) was for diagnosis and the remaining was for treatment. The programmatic cost for the non-TB cohort was estimated at $11 million (95% UI: 4 to 25) with $5 million (95% UI: 1 to 17) for diagnosis and the remaining attributable to treatment of false-positive cases.

### Health losses along the TB care cascade

Table 2 reports the total TB-attributable deaths, DALYs, and costs estimated for a set of alternative scenarios that assume perfect programmatic performance at a given step of the care cascade. We compared these values to current program performance to estimate the relative magnitude of health losses for each cascade step. The greatest source of estimated health losses along the TB care cascade was delayed TB diagnosis, with DALYs and deaths from TB reduced by 71.1% (95% UI: 65.1 to 76.2) and 63.5% (95% UI: 56.4 to 69.4), respectively, in a scenario that eliminated delays before TB diagnosis. Eliminating post-TB sequelae produced the next largest reduction in DALYs (41.4% (95% UI: 35.8 to 48.8)), followed by loss to follow-up from TB treatment (10.0% (95% UI: 6.7 to 15.5)). Elimination of loss to follow-up from TB treatment was estimated to reduce TB deaths by 12.7% (95% UI: 8.6 to 18.5). The greatest reduction in total person-years spent with infectious TB was obtained by removing delays to diagnosis and treatment loss to follow-up with reductions of 52.2% (95% UI: 42.9 to 58.9) and 15.6% (95% UI: 10.9 to 22.8) in those scenarios, respectively. The fraction cured with early TB was significantly higher with removal of delays to diagnosis with 84.0% (95% UI: 80.5 to 86.7) curing with early TB compared to 28.7% (95% UI: 20.8 to 39.6) at baseline.

**Table 2:** Change in deaths with TB, TB-attributable DALYs, and TB programmatic costs with elimination of gaps in the care cascade, as compared with current program performance in Brazil.

Scenario	Total TB deaths		Total TB attributable DALYs		Total TB programmatic costs		Transmission burden		Percent cured with early TB
	Value	Percent change	Value (thousands)	Percent change	Value (millions USD)	Percent change	Value (thousands yrs)	Percent change	Value (%)
***Reference scenario***									
Current programmatic performance	7,240 (5,760, 8,570)	-----	254 (207, 310)	-----	107 (86, 132)	-----	47 (40, 57)	-----	28.7 (20.8, 39.6)
***Alternative scenarios***									
No delays to diagnosis	2,650 (1,960, 3,480)	−63.5 (−69.4, −56.4)	73 (55, 94)	−71.1 (−76.2, −65.1)	108 (89, 131)	1.3 (−6.7, 7.2)	22 (18, 29)	−52.2 (−58.9, −42.9)	84.0 (80.5, 86.7)
No false negative diagnoses	6,950 (5,590, 8,370)	−4.0 (−6.5, −1.9)	245 (202, 283)	−3.6 (−5.8, −1.7)	106 (86, 131)	−0.8 (−1.8, −0.1)	44 (38, 54)	−5.0 (−7.8, −2.5)	30.7 (22.6, 41.5)
No false-positive diagnoses	7,240 (5,760, 8,570)	-----	253 (210, 293)	−0.1 (−0.1, 0.0)	102 (80, 120)	−5.0 (−10.2, −1.6)	47 (40, 57)	-----	28.7 (20.8, 39.6)
Rifampicin resistance identified at initial diagnosis	7,180 (5,730, 8,520)	−0.8 (−1.4, −0.4)	252 (209, 292)	−0.6 (−1.0, −0.3)	106 (85, 131)	−0.5 (−1.6, 0.8)	46 (40, 57)	−0.8 (−1.3, −0.5)	28.8 (20.9, 39.7)
No pretreatment loss to follow up	7,140 (5,690, 8,450)	−1.5 (−1.9, −1.1)	250 (208, 290)	−1.3 (−1.7, −0.9)	107 (86, 131)	−0.3 (−0.5, 0.0)	46 (39, 56)	−1.8 (−2.3, −1.4)	29.4 (21.5, 40.3)
No treatment loss to follow up	6,320 (5,050, 7,640)	−12.7 (−18.5, −8.6)	228 (188, 266)	−10.0 (−15.5, −6.7)	106 (86, 132)	−0.4 (−2.6, 1.6)	39 (33, 47)	−15.6 (−22.8, −10.9)	32.0 (23.3, 44.0)
No treatment failure	6,930 (5,570, 8,290)	−4.4 (−8.0, −1.8)	246 (204, 284)	−3.1 (−5.7, −1.3)	104 (82, 126)	−3.1 (−5.4, −1.5)	45 (39, 54)	−3.0 (−5.8, −1.2)	29.3 (21.2, 40.1)
No delay in retreatment after treatment failure	7,060 (5,650, 8,370)	−2.5 (−4.3, −1.3)	249 (205, 287)	−2.0 (−3.4, −1.1)	108 (87, 133)	1.4 (0.5, 2.4)	45 (39, 54)	−3.6 (−6.1, −1.9)	29.3 (21.3, 40.2)
No post-TB sequelae after TB cure	7,240 (5,760, 8,570)	-----	149 (116, 178)	−41.4 (−48.8, −35.8)	107 (86, 132)	-----	47 (40, 57)	-----	28.7 (20.8, 39.6)

DALY, disability-adjusted life year; TB, tuberculosis; USD, United States dollars.

The programmatic costs associated with each scenario are shown in Table 2. While each alternative scenario was estimated to produce health improvements compared to the base case, the effect on costs was less consistent. Eliminating false-positive diagnoses produced the greatest cost reduction (5.0% [95% UI: 1.6 to 10.2]), while eliminating treatment failure reduced costs by 3.1% (95% UI: 1.5 to 5.4). In contrast, eliminating diagnostic delays increased costs on average by 1.3% (95% UI: −6.7 to 7.2), due to greater numbers presenting for diagnosis and initiating treatment, although this effect was highly sensitive to parametric variation.

Scenario analyses for cohorts with HIV coinfection and RR-TB are given in S4 Table.

### Sensitivity analyses

S7 Fig shows the impact of parametric uncertainty on model outcomes. TB-attributable DALYs were most sensitive to TB mortality rates, rates of progression from early to late TB, and rates of presentation for diagnosis for individuals with late TB. Sensitivity analysis for case fatality and programmatic cost outcomes as well as non-TB cohort model sensitivities are also shown in S7 Fig.

## Discussion

In this study, we examined the performance of the care cascade for TB in Brazil and examined the health losses and costs attributable to gaps at each step of this cascade. We first present a thorough analysis of the current program performance detailing estimates of TB burden and health system costs stratified by age, state, HIV status, rifampin resistance status and early versus late disease status. We then perform scenario analyses that indicate that delayed case detection and post-TB sequelae are primary sources of TB-attributable disease burden in Brazil, followed by treatment loss to follow-up. These findings are consistent with the results of care cascade analyses in other countries [16].

Our study adds to existing literature by highlighting an understudied contributor to TB disease burden—post-TB sequelae among TB survivors. Our model estimates that post-TB sequelae produce 41% of TB-attributable DALYs due to increases in mortality and morbidity [9]. Moreover, the large contribution to DALYs reduction caused by removal of post-TB sequelae suggests that the benefit of early diagnosis, which increases the fraction cured with early TB from 29% to 84%, may be substantially mediated by reductions of cured late TB sequelae. The inclusion of this factor in the model reflects recent research demonstrating the range and magnitude of post-TB lung disease [31].

Individuals with TB-HIV and RR-TB were estimated to experience elevated TB mortality and DALYs relative to individuals without these factors. While for HIV-infected individuals, closing gaps in the care cascade was estimated to produce health improvements approximately proportional to those estimated for the overall TB cohort, the effect sizes were smaller for individuals with RR-TB. For example, eliminating delays to care produced a 26% reduction in DALYs in the RR-TB cohort, as compared to 71% for the overall cohort. This finding could result from the poorer treatment outcomes of RR-TB even when treatment is initiated promptly, highlighting the challenges of achieving successful treatment outcomes for these individuals. Recently published expedited regimens for treatment of RR-TB may reduce deficits caused by increased length of disease and overall probability of loss to follow-up [32].

While closing gaps in the care cascade was estimated to produce health benefits, these scenarios produced relatively small changes in TB program costs. Eliminating treatment failure was estimated to reduce costs by 3%, while all other changes for the TB cohort either had minimal effects or increased the costs of care. For the non-TB cohort, we estimated that misdiagnosis with TB (false-positive diagnoses) contributed a relative negligible fraction of overall DALY estimates. However, the costs associated with diagnosis and treatment of individuals without TB presenting with TB-like symptoms constituted 10% of total programmatic costs. Of this, the costs of incorrectly treating individuals without TB represented 2% of total programmatic costs, indicating that TB misdiagnosis accounts for a large portion of reducible programmatic costs.

The results estimated for current programmatic performance indicate that individuals with TB-HIV and RR-TB experience 3 times the case fatality as the overall TB cohort and experience 1.4 and 2.0 times the per person TB DALYs, respectively, reflecting the poorer TB disease outcomes for these individuals. The TB burden of disease by age strata demonstrate that the aggregate TB burden is highest for individuals aged 20 to 45 years, while per-person DALYs are highest among infants with TB, due to both elevated mortality rates and greater numbers of life years lost with each death in this age group.

The findings of this analysis have several implications for policy. First, despite Brazil’s efforts to achieve universal health coverage—including the provision of free publicly funded TB care—many individuals who develop TB experience substantial morbidity and mortality risks from the disease, stemming from gaps and delays in the care cascade. Closing these gaps could yield major health improvements. Second, these results demonstrate the central importance of accelerating case detection for improving TB outcomes. Doing so would not only reduce pretreatment morbidity and deaths, but also reduce the post-TB sequelae among individuals surviving the disease and reduce transmission risks. Expanded healthcare access and deployment of higher-sensitivity diagnostics could help reduce the gaps in TB case detection. Third, this analysis shows the health system benefits of improved treatment completion and adherence, as well as subsequent rehabilitative services for individuals with persistent post-TB sequelae. Finally, the analysis motivates improved implementation of high-fidelity, low-cost diagnostic testing to reduce TB programmatic costs associated with diagnosis and treatment of false-positive cases. Additional analyses are required to understand how Brazil should prioritize different intervention options to minimize the health impact of TB, considering not only the consequences at each stage of the TB care cascade (as quantified in this analysis), but also the costs of introducing each intervention, and the likely barriers to doing so.

The analysis reports strong calibration and validation metrics, giving credibility to a modeling approach that included stratification of HIV status, drug resistance status, age, state of residence and progression of TB disease. However, this analysis has several limitations. The analysis relied heavily on treatment initiation and mortality data, which may introduce bias for a variety of factors (e.g., decreased mortality reporting for minorities). Moreover, official TB program data (SINAN) may misrepresent some outcomes—such as losses in the care cascade caused by loss to follow-up and diagnostic error—which may be better captured by other data sources. Additional strata such as sex, social protection status, and history of incarceration were not included in the model to limit complexity, although they are known to impact access to TB care and health outcomes. Similarly, we took a simplified approach to modeling ART coverage among individuals with HIV (only considering receipt of ART at the point of TB diagnosis) and did not model HIV viral dynamics or ART initiation/discontinuation. The stages of disease (early versus late) were modeled as a discrete transition as opposed to a continuous continuum of disease severity. While the consequences of disease transmission and secondary cases were estimated using changes in duration of infectious disease, this is an approximation of the change in transmission patterns that would occur under the analytic scenarios, which would require a transmission dynamic model to capture accurately. Finally, this model was calibrated to data acquired before the COVID-19 pandemic, which altered the subsequent TB epidemiology and health services. Further analyses should estimate care cascade performance following the pandemic.

Overall, this analysis provides a baseline framework for evaluating the costs and benefits of possible TB program improvements across state and demographic strata in Brazil. While the analysis was not structured to analyze specific health interventions, our results indicate that intervention strategies that can effectively reduce delays to diagnosis, reduce treatment loss to follow-up, and achieve effective rehabilitation of post-TB sequelae may have the greatest impact on TB disease burden. Further analyses are needed to estimate the potential impact and cost-effectiveness of possible intervention strategies to guide the direction of TB services in Brazil.

## Supporting information

S1 Fig Detailed dynamics of TB cohort model, showing stratification by early (A, B) vs. late (C, D) disease and RS-TB (A, C) vs. RR-TB (B, D). Transitions to the death state are omitted and implied from every depicted health state in the TB cohort. 1L/2L: first-line/second-line, DST: drug sensitivity testing; LTFU: loss to follow-up, RR: rifampin resistant, TB: tuberculosis.
(TIFF)

S2 Fig Estimated and calibrated mortality rates of TB disease states. ART: antiretroviral therapy, HIV: human immunodeficiency virus, TB: tuberculosis.
(TIFF)

S3 Fig Calibration performance of model against SIM deaths by state (A) and SINAN deaths by age and HIV (B). HIV: human immunodeficiency virus, TB: tuberculosis.
(TIFF)

S4 Fig Comparison of one-year case fatality predicted by the model against linked-cohort validation dataset for HIV unaffected (A) and HIV affected (B) individuals.
(TIFF)

S5 Fig Estimated incidence rate (A) and total incidence by state (B) and age (C), compared with estimates from Chitwood and colleagues. TB: tuberculosis.
(TIFF)

S6 Fig Dynamics of health state transitions within the care cascade over the full patient lifespan (A) and over a relevant 2-year TB disease interval (B). TB: tuberculosis.
(TIFF)

S7 Fig Multivariable sensitivity analyses using a sample of 1,000 parameters for TB cohort model outcomes—per person DALYs (A), case fatality (B), and per person costs (C). Sensitivity analysis for non-TB cohort model outcomes—per person DALYs (D) and per person costs. Univariate sensitivity analyses for total (both TB and non-TB cohorts) DALYs (F) and health system costs (G). DALY: disability-adjusted life year, HIV: human immunodeficiency virus, RR/RS: rifampin resistant/sensitive, TB: tuberculosis, USD: United States dollars.
(TIFF)

S1 Table Parameter table. *Parameters based on assumption were determined through discussion between study investigators and Ministry of Health staff and informed with programmatic data where available. HIV: human immunodeficiency virus, RS/RR: rifampin-susceptible/resistant, TB: tuberculosis.
(DOCX)

S2 Table Estimated loss to follow-up rate, primary loss to follow-up fraction, and rate of presentation for TB diagnosis by state. (DOCX)

S3 Table Description of modeled health system scenarios and associated parametric adjustments. TB: tuberculosis. (DOCX)

S4 Table Scenario-based burden of disease assessment for subgroups of cases with HIV coinfection (A) and RR-TB (B). Parentheses indicate 95% UI in simulations of parametric uncertainty. TB: tuberculosis.
(DOCX)

S1 TRIPOD Checklist Prediction model development. (PDF)

## Abbreviations

ART: antiretroviral therapy
COVID-19: Coronavirus Disease 2019
DALY: disability adjusted life year
HIV: human immunodeficiency virus
PRCC: partial rank correlation coefficient
RR-TB: rifampicin-resistant tuberculosis
TB: tuberculosis
UI: uncertainty interval
YLD: year lived with disability
YLL: year of life los
