# Supplementary figures and images for: Quantifying gaps in the tuberculosis care cascade in Brazil: A mathematical model study using national program data
Source: PLoS Med. 2024 Mar 21;21(3):e1004361. doi: 10.1371/journal.pmed.1004361 (PMC10994550; doi:10.1371/journal.pmed.1004361)

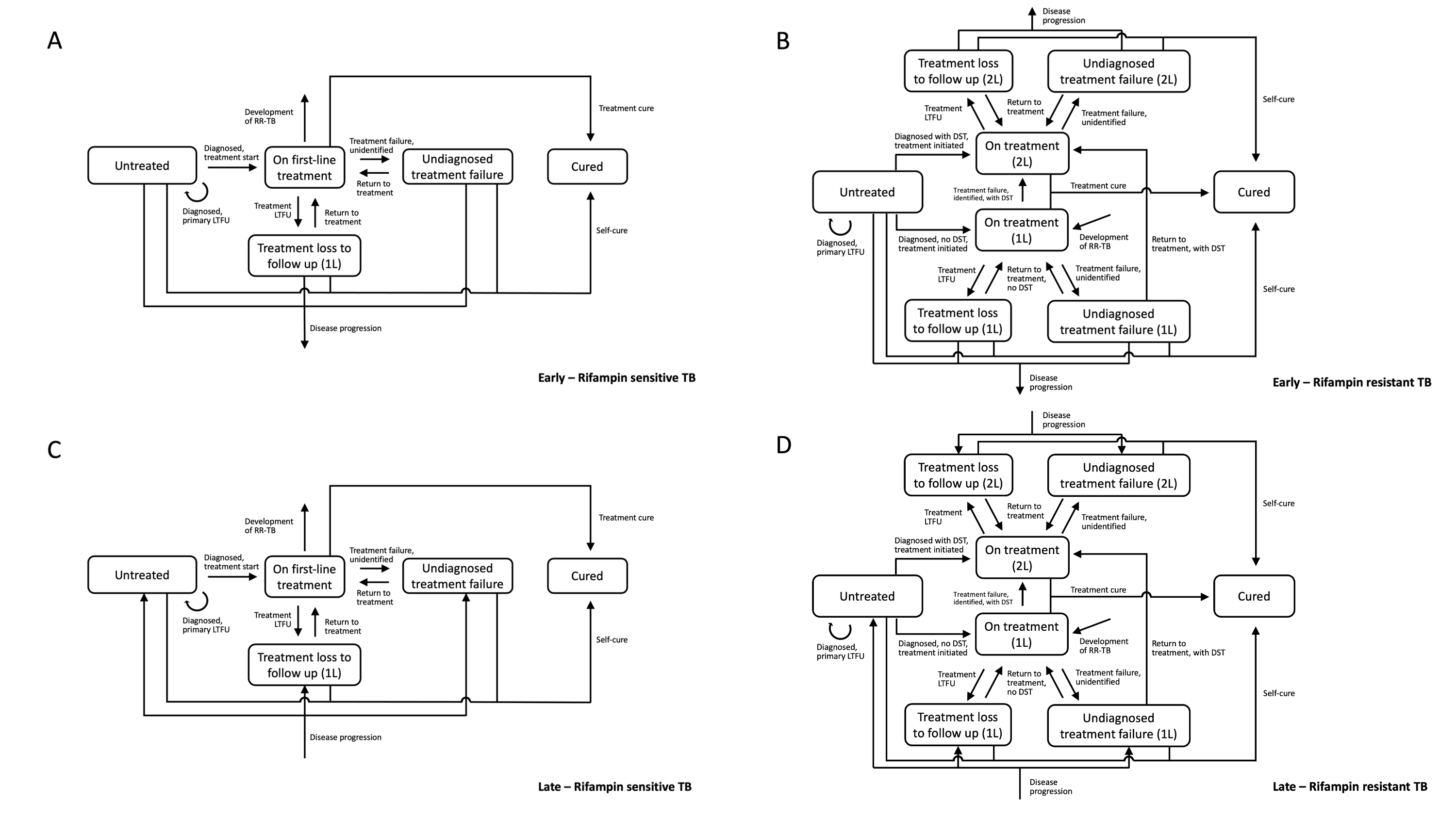

Supplement: S1 Fig — Detailed dynamics of TB cohort model, showing stratification by early (A, B) vs. late (C, D) disease and RS-TB (A, C) vs. RR-TB (B, D). Transitions to the death state are omitted and implied from every depicted health state in the TB cohort. 1L/2L: first-line/second-line, DST: drug sensitivity testing; LTFU: loss to follow-up, RR: rifampin resistant, TB: tuberculosis. (TIFF) [file pmed.1004361.s001.tiff]

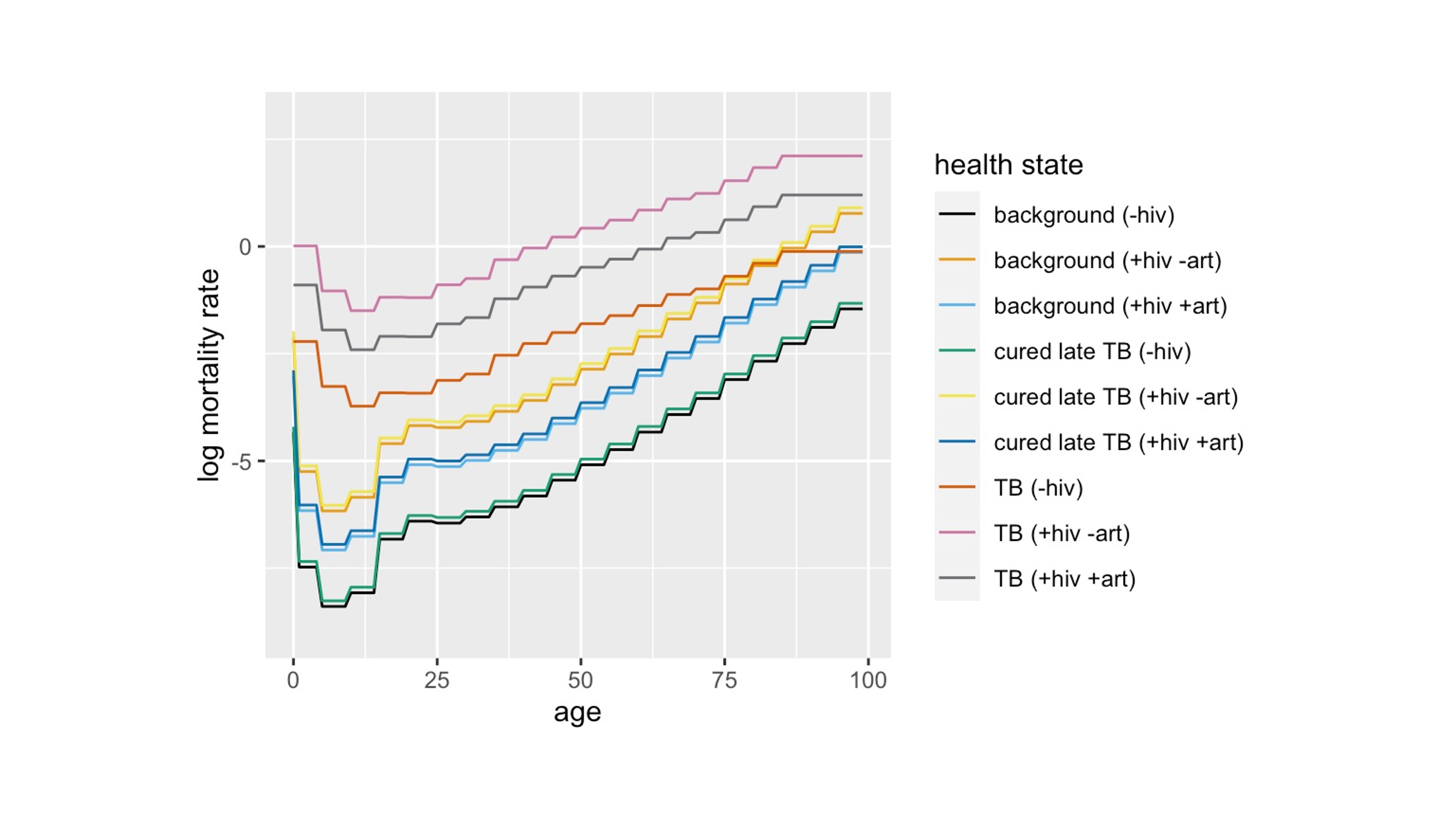

Supplement: S2 Fig — ART: antiretroviral therapy, HIV: human immunodeficiency virus, TB: tuberculosis. (TIFF) [file pmed.1004361.s002.tiff]

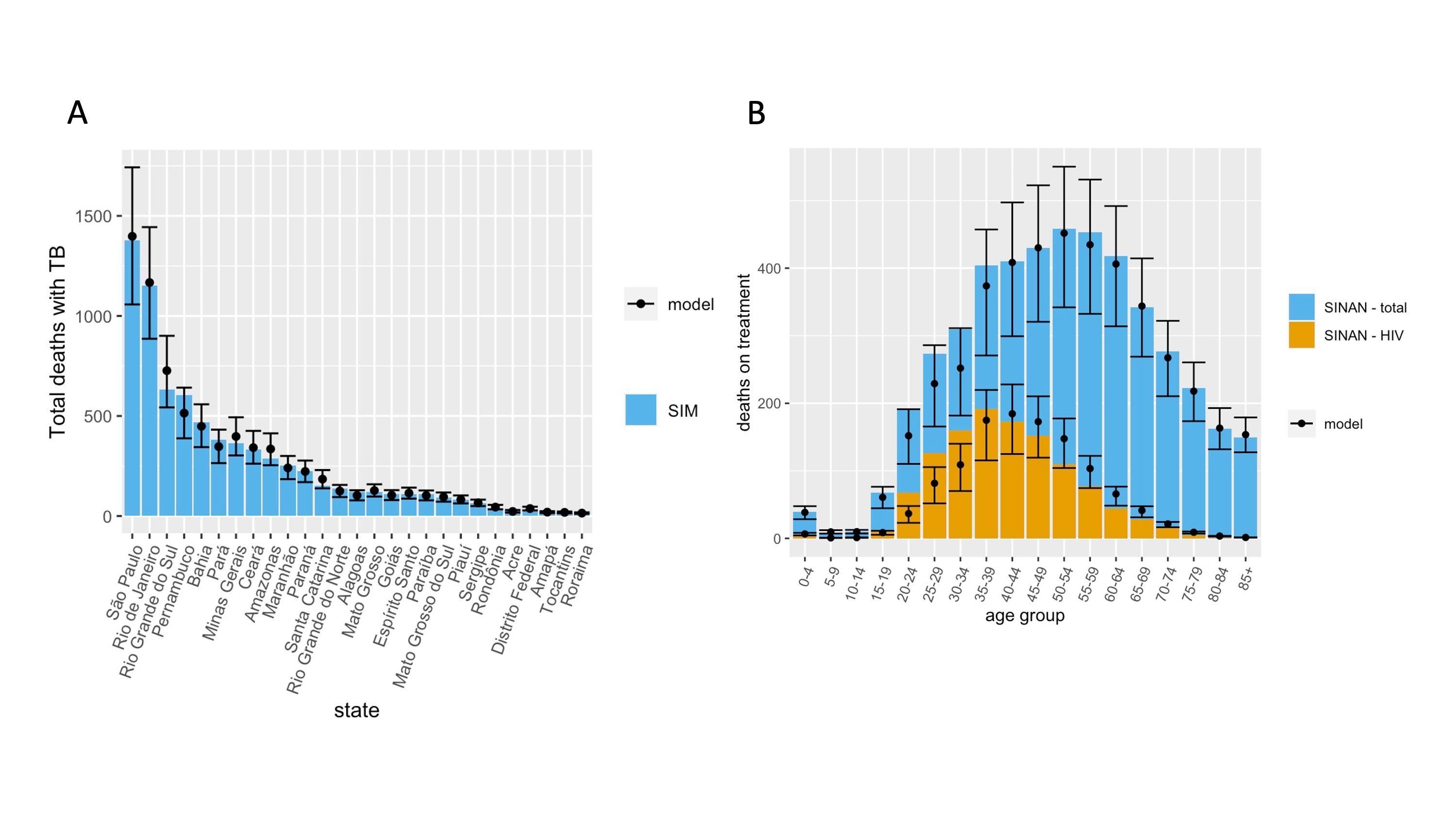

Supplement: S3 Fig — Calibration performance of model against SIM deaths by state (A) and SINAN deaths by age and HIV (B). HIV: human immunodeficiency virus, TB: tuberculosis. (TIFF) [file pmed.1004361.s003.tiff]

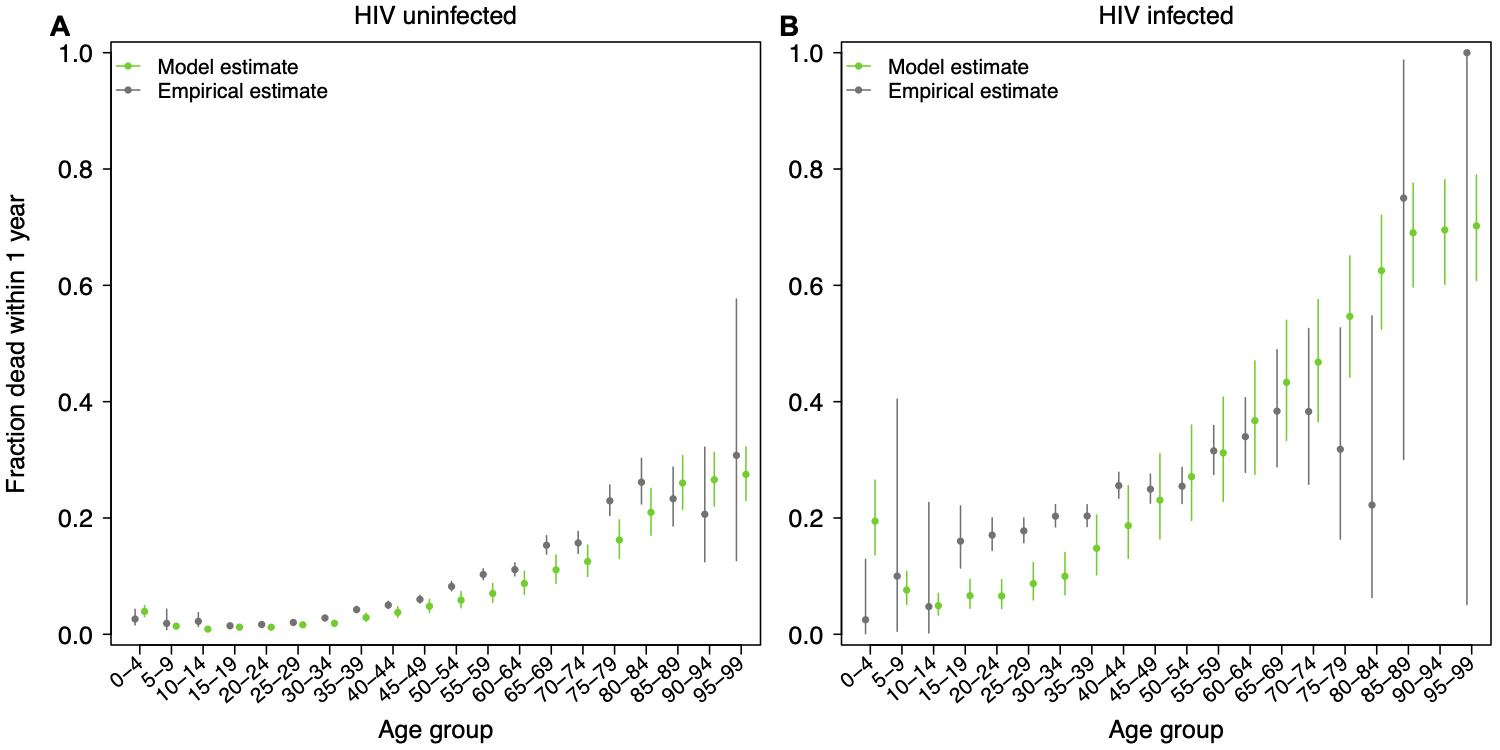

Supplement: S4 Fig — Comparison of one-year case fatality predicted by the model against linked-cohort validation dataset for HIV unaffected (A) and HIV affected (B) individuals. (TIFF) [file pmed.1004361.s004.tiff]

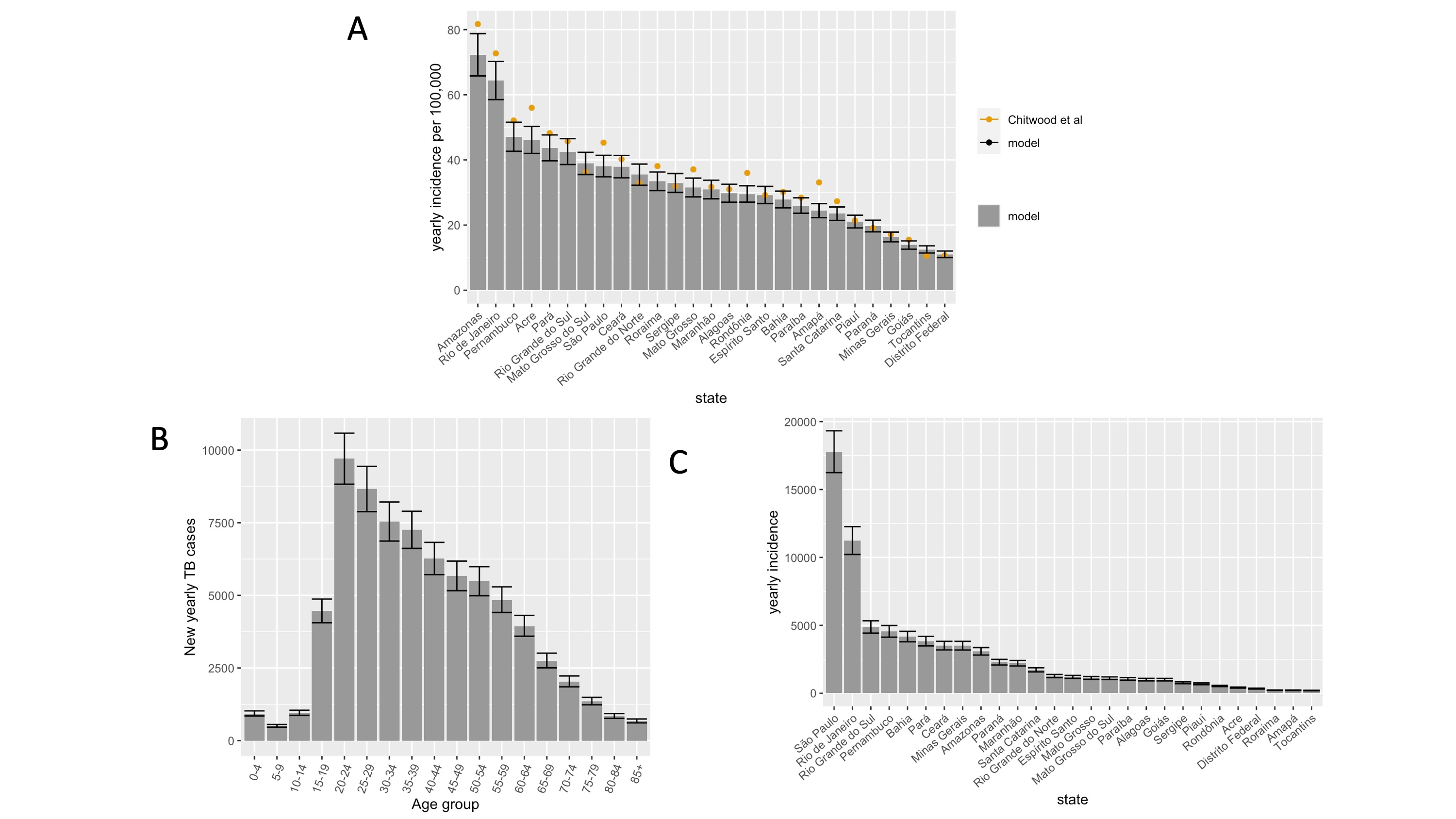

Supplement: S5 Fig — Estimated incidence rate (A) and total incidence by state (B) and age (C), compared with estimates from Chitwood and colleagues. TB: tuberculosis. (TIFF) [file pmed.1004361.s005.tiff]

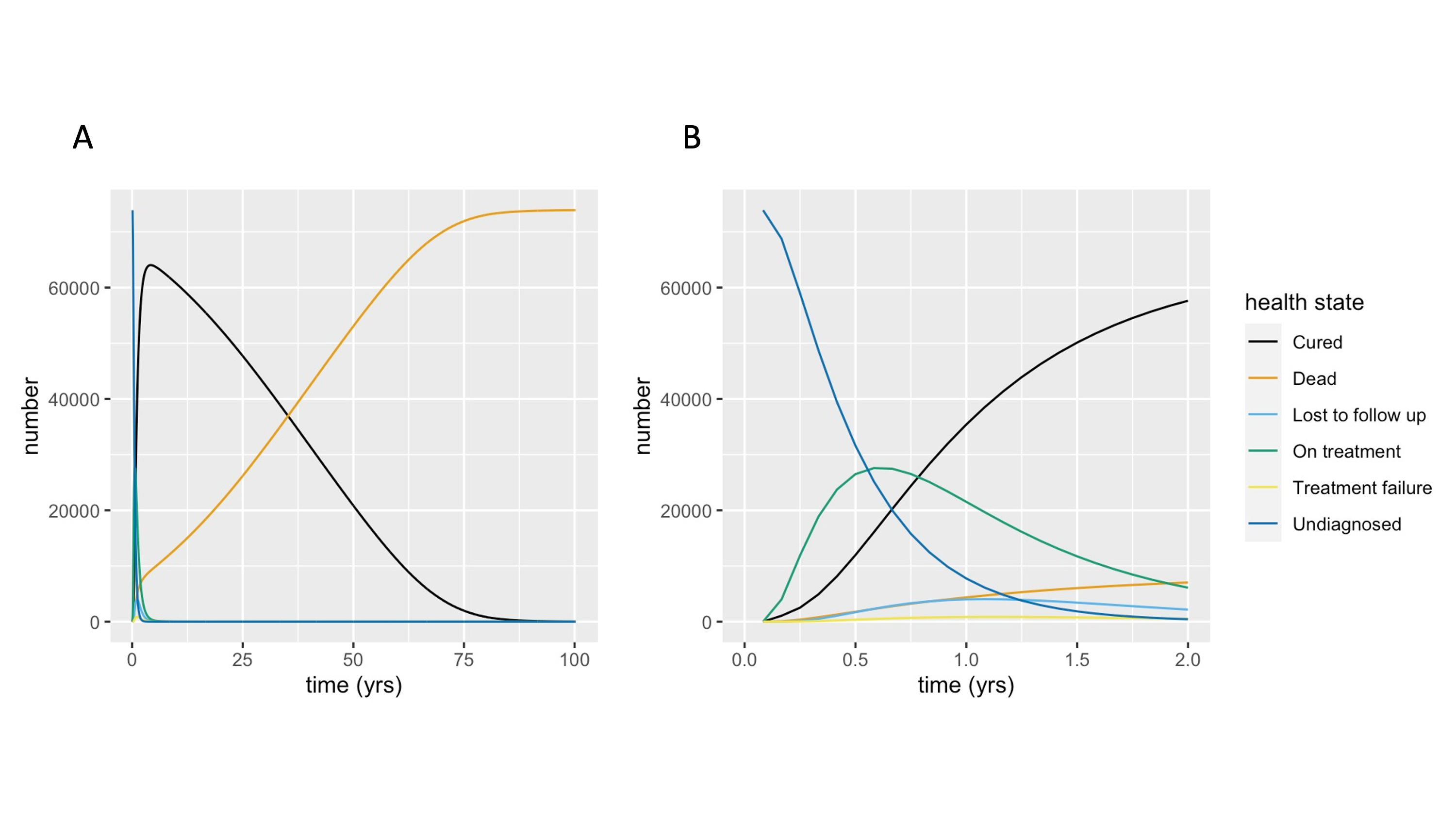

Supplement: S6 Fig — Dynamics of health state transitions within the care cascade over the full patient lifespan (A) and over a relevant 2-year TB disease interval (B). TB: tuberculosis. (TIFF) [file pmed.1004361.s006.tiff]

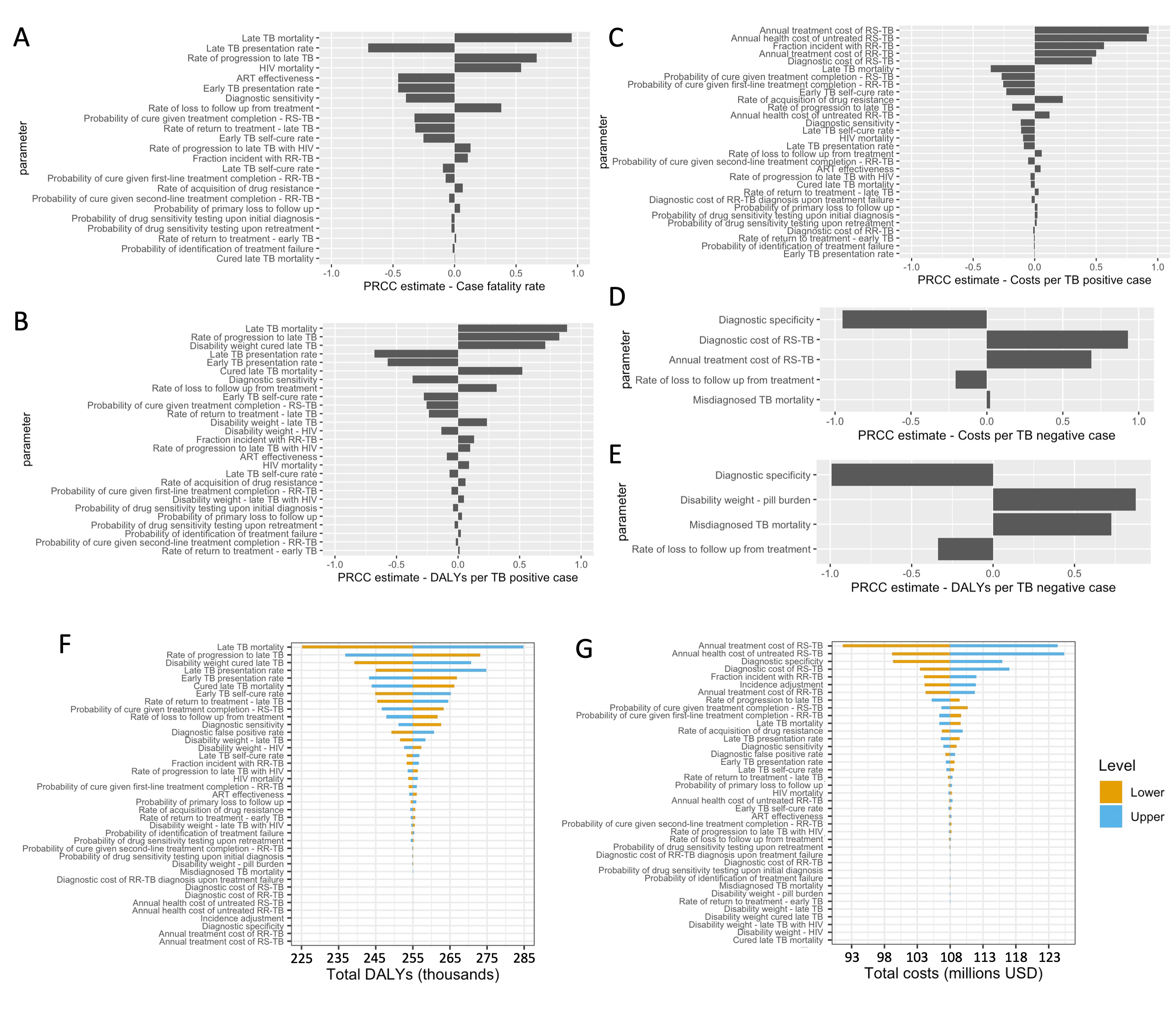

Supplement: S7 Fig — Multivariable sensitivity analyses using a sample of 1,000 parameters for TB cohort model outcomes—per person DALYs (A), case fatality (B), and per person costs (C). Sensitivity analysis for non-TB cohort model outcomes—per person DALYs (D) and per person costs. Univariate sensitivity analyses for total (both TB and non-TB cohorts) DALYs (F) and health system costs (G). DALY: disability-adjusted life year, HIV: human immunodeficiency virus, RR/RS: rifampin resistant/sensitive, TB: tuberculosis, USD: United States dollars. (TIFF) [file pmed.1004361.s007.tiff]
