# Supplementary material for: Quantifying gaps in the tuberculosis care cascade in Brazil: A mathematical model study using national program data
Source: PLoS Med. 2024 Mar 21;21(3):e1004361. doi: 10.1371/journal.pmed.1004361 (PMC10994550; doi:10.1371/journal.pmed.1004361)
Supplement: S1 Table — *Parameters based on assumption were determined through discussion between study investigators and Ministry of Health staff and informed with programmatic data where available. HIV: human immunodeficiency virus, RS/RR: rifampin-susceptible/resistant, TB: tuberculosis. (DOCX) [file pmed.1004361.s008.docx]

S1 Table. Parameter table

| **Parameter** | **Value (95% uncertainty interval)** | **Source** | **In PSA?** |
| --- | --- | --- | --- |
| *Cohort generation* | | | |
| Fraction of incident cohort with RR-TB | 0.016 (0.010 - 0.022) | WHO 2022 [1] | Yes |
| Fraction of recorded SINAN deaths that are true deaths | 0.71 | Chitwood 2022 [2] | No |
| Additional uncertainty in total TB incidence | 1 (0.96 – 1.04) | Annual variation in SINAN treatment notifications 2017 – 2019 | Yes |
| *Mortality* | | | |
| Background mortality rate (per year) | see S2 Fig | UN Pop Division 2023 [3] | No |
| Mortality rate for individuals with late TB (vs. background) | see S2 Fig | Base rate calibrated to available data; age-dependent adjustment from SINAN | Yes (base rate) |
| Mortality rate ratio of HIV (vs. no HIV) | 4.54 (3.40 – 5.67) | Calibrated to available data | Yes |
| Mortality rate ratio for cured late TB (vs. background) | 1.14 (1.10 – 1.18) | Menzies 2021 [4] | Yes |
| Mortality rate ratio for TB treatment following false-positive diagnosis (vs. background) | 1.07 (1.02 – 1.13) | Blount 2010 [5] | Yes |
| *Cure/treatment failure* | | | |
| Rate of self-cure with late TB (per year) | 0.10 (0.05 – 0.15) | Tiemersma 2011 [6] | Yes |
| Rate ratio of self-cure with early TB, compared with late TB | 2.0 (1.5 – 2.5) | Assumption* | Yes |
| Rate of treatment completion for first line regimen (per year) | 2.0 | Assumed 6-month treatment duration | No |
| Rate of treatment completion for second line regimen (per year) | 0.67 | Assumed 18-month treatment duration [7] | No |
| Probability of cure for individuals with RS-TB completing the first-line regimen | 0.96 (0.92 – 0.98) | Cox 2008 [8] | Yes |
| Risk ratio of cure for individuals with RR-TB completing the first-line regimen (vs. RS-TB receiving first-line regimen) | 0.20 (0.10 - 0.30) | Miglori 2002 [9] |  |
| Risk ratio of cure for individuals with RR-TB completing the second-line regimen (vs. RS-TB receiving first-line regimen) | 0.93 (0.89 - 0.96) | Miglori 2002 [9] | Yes |
| Rate of return to treatment from undiagnosed treatment failure (late TB) (per year) | 2.53 (1.27 - 3.80) | Maior 2012 [10] | Yes |
| Rate ratio of return to treatment from undiagnosed treatment failure (early TB), compared with late TB | 0.100 (0.075 - 0.125) | Assumption* | Yes |
| Probability of identifying treatment failure given treatment completion without cure | 0.10 (0.05 - 0.20) | SINAN | Yes |
| *Diagnosis* | | | |
| Rate of presentation for diagnosis with undiagnosed late TB (per year) | see S2 Table | Calibrated to available data | Yes (ratio from baseline) |
| Rate ratio of presentation for diagnosis with undiagnosed early TB, compared with late TB | 0.100 (0.075 - 0.125) | Assumption* | Yes |
| Sensitivity of diagnostic testing | 0.87 (0.80 - 0.95) | Derived from sensitivity, specificity, and fraction of SINAN cases with clinical vs bacteriologic testing | Yes |
| Specificity of diagnostic testing | 0.92 (0.85 - 0.99) | Derived from sensitivity, specificity, and fraction of SINAN cases with clinical vs bacteriologic testing | Yes |
| Fraction of TB treatment cohort with no TB (misdiagnosed) | 0.095 (0.033 - 0.157) | Assumption* | Yes |
| *Drug resistance* | | | |
| Probability of testing for drug resistance upon TB diagnosis | 0.30 (0.25 - 0.35) | SINAN | Yes |
| Probability of testing for drug resistance on TB retreatment | 0.38 (0.32 – 0.44) | SINAN | Yes |
| Rate of drug resistance acquisition on first line TB treatment (per year) | 0.010 (0.006 - 0.016) | Menzies 2012 [11] | Yes |
| *Loss to follow up* | | | |
| Probability of loss to follow up between TB diagnosis and treatment initiation (primary loss to follow-up) | 0.05 (0.04 - 0.06) national average; see S2 Table for state-level estimates | Assumption* (national average), SINAN loss to follow-up (state-level) | Yes |
| Rate of loss to follow up from TB treatment (per year) | See S2 Table | SINAN | Yes |
| Rate of return from loss to follow up (late TB) to treatment (per year) | 2.53 (1.27 - 3.80) | Maior 2012 [10] | Yes |
| Rate ratio of return from loss to follow up (early TB), compared with late TB | 0.100 (0.075 - 0.125) | Assumption* | Yes |
| *Progression* | | | |
| Rate of disease progression from early to late TB with HIV negative TB (per year) | 2.0 (1.5 - 3.0) | Ku 2021 [12] | Yes |
| Rate of disease progression from early to late TB with HIV positive TB (per year) | 9.0 (4.5 - 13.5) | Ku 2021 [12] | Yes |
| *Disability weights* | | | |
| Disability weight for HIV negative TB | 0.333 (0.250 - 0.417) | Salomon 2015 [13] | Yes |
| Disability weight for HIV positive TB | 0.400 (0.300 - 0.500) | Salomon 2015 [13] |  |
| Disability weight for HIV (no TB) | 0.200 (0.150 - 0.250) | Salomon 2015 [13] | Yes |
| Disability weight for cured late TB | 0.036 (0.027 - 0.045) | Salomon 2015 [13] | Yes |
| Disability weight for TB treatment for TB negative patient | 0.049 (0.036 - 0.060) | Salomon 2015 [13] | Yes |
| *Costs* | | | |
| Cost of TB diagnosis | 54.3 (27.2 - 108.7) | Nsengiyumva 2022 [14] | Yes |
| Diagnostic cost for RR-TB from treatment failure state | 16.6 (8.4 - 33.4) | Nsengiyumva 2022 [14] | Yes |
| Monthly treatment cost for RS-TB | 144 (108 - 180) | Nsengiyumva 2022 [14] | Yes |
| Monthly treatment cost for RR-TB | 619 (464 - 773) | Nsengiyumva 2022 [14] | Yes |
| Monthly primary care cost for untreated TB | 33.4 (16.7 - 66.8) | Nsengiyumva 2022 [14] | Yes |

* Parameters based on assumption were determined through discussion between study investigators and Ministry of Health staff and informed with programmatic data where available. HIV: Human Immunodeficiency Virus, RS/RR: rifampin-susceptible/resistant, TB: Tuberculosis

References

1. Organization WH. Global Tuberculosis Report 2022. Geneva: World Health Organization. 2022.

2. Chitwood MH, Alves LC, Bartholomay P, Couto RM, Sanchez M, Castro MC, et al. A spatial-mechanistic model to estimate subnational tuberculosis burden with routinely collected data: An application in Brazilian municipalities. PLOS Glob Public Health. 2022;2(9):e0000725. Epub 20220921. doi: 10.1371/journal.pgph.0000725. PubMed PMID: 36962578; PubMed Central PMCID: PMCPMC10021638.

3. World Population Prospects 2022 [Internet]. United Nations Population Division, Department of Economic and Social Affairs. 2022.

4. Menzies NA, Quaife M, Allwood BW, Byrne AL, Coussens AK, Harries AD, et al. Lifetime burden of disease due to incident tuberculosis: a global reappraisal including post-tuberculosis sequelae. Lancet Glob Health. 2021;9(12):e1679-e87. doi: 10.1016/S2214-109X(21)00367-3. PubMed PMID: 34798027; PubMed Central PMCID: PMCPMC8609280.

5 . Blount RJD, A.; Cattamanchi, L.; Worodria, W.; den Boon, S.; Joloba, M.; Huang, L. The Effect Of False Positive And False Negative Microscopy Results On Mortality Among HIV-infected Ugandans Undergoing Evaluation For TB. American Thoracic Society: American Journal of Respiratory Critical Care Medicine; 2010.

6. Tiemersma EW, van der Werf MJ, Borgdorff MW, Williams BG, Nagelkerke NJ. Natural history of tuberculosis: duration and fatality of untreated pulmonary tuberculosis in HIV negative patients: a systematic review. PLoS One. 2011;6(4):e17601. Epub 20110404. doi: 10.1371/journal.pone.0017601. PubMed PMID: 21483732; PubMed Central PMCID: PMCPMC3070694.

7. WHO consolidated guidelines on tuberculosis: Module 4: treatment - drug-resistant tuberculosis treatment, 2022 update. WHO Guidelines Approved by the Guidelines Review Committee. Geneva2022.

8. Cox HS, Morrow M, Deutschmann PW. Long term efficacy of DOTS regimens for tuberculosis: systematic review. BMJ. 2008;336(7642):484-7. Epub 20080204. doi: 10.1136/bmj.39463.640787.BE. PubMed PMID: 18250104; PubMed Central PMCID: PMCPMC2258398.

9. Migliori GB, Espinal M, Danilova ID, Punga VV, Grzemska M, Raviglione MC. Frequency of recurrence among MDR-tB cases 'successfully' treated with standardised short-course chemotherapy. Int J Tuberc Lung Dis. 2002;6(10):858-64. PubMed PMID: 12365571.

10. Maior Mde L, Guerra RL, Cailleaux-Cezar M, Golub JE, Conde MB. Time from symptom onset to the initiation of treatment of pulmonary tuberculosis in a city with a high incidence of the disease. J Bras Pneumol. 2012;38(2):202-9. doi: 10.1590/s1806-37132012000200009. PubMed PMID: 22576428; PubMed Central PMCID: PMCPMC3697913.

11. Menzies NA, Cohen T, Lin HH, Murray M, Salomon JA. Population health impact and cost-effectiveness of tuberculosis diagnosis with Xpert MTB/RIF: a dynamic simulation and economic evaluation. PLoS Med. 2012;9(11):e1001347. Epub 20121120. doi: 10.1371/journal.pmed.1001347. PubMed PMID: 23185139; PubMed Central PMCID: PMCPMC3502465.

12. Ku C-C, MacPherson P, Khundi M, Nzawa Soko RH, Feasey HRA, Nliwasa M, et al. Durations of asymptomatic, symptomatic, and care-seeking phases of tuberculosis disease with a Bayesian analysis of prevalence survey and notification data. BMC Medicine. 2021;19(1):298. doi: 10.1186/s12916-021-02128-9.

13. Salomon JA, Haagsma JA, Davis A, de Noordhout CM, Polinder S, Havelaar AH, et al. Disability weights for the Global Burden of Disease 2013 study. Lancet Glob Health. 2015;3(11):e712-23. doi: 10.1016/S2214-109X(15)00069-8. PubMed PMID: 26475018.

14 Nsengiyumva NP, Campbell JR, Oxlade O, Vesga JF, Lienhardt C, Trajman A, et al. Scaling up target regimens for tuberculosis preventive treatment in Brazil and South Africa: An analysis of costs and cost-effectiveness. PLoS Med. 2022;19(6):e1004032. Epub 20220613. doi: 10.1371/journal.pmed.1004032. PubMed PMID: 35696431; PubMed Central PMCID: PMCPMC9239450.
