# Supplementary material for: Quantifying gaps in the tuberculosis care cascade in Brazil: A mathematical model study using national program data
Source: PLoS Med. 2024 Mar 21;21(3):e1004361. doi: 10.1371/journal.pmed.1004361 (PMC10994550; doi:10.1371/journal.pmed.1004361)
Supplement: S2 Table — (DOCX) [file pmed.1004361.s009.docx]

**Table S4.** Estimated loss to follow-up rate, primary loss to follow-up fraction and rate of presentation for TB diagnosis by state.

| **State** | **Loss to follow up rate** | **Primary loss to follow up fraction** | **Rate of presentation for diagnosis** |
| --- | --- | --- | --- |
| Acre | 0.086 | 0.014 | 10.1 |
| Alagoas | 0.388 | 0.032 | 6.7 |
| Amapá | 0.294 | 0.040 | 10.8 |
| Amazonas | 0.550 | 0.062 | 9.2 |
| Bahia | 0.383 | 0.040 | 7.2 |
| Ceará | 0.498 | 0.055 | 9.0 |
| Distrito Federal | 0.417 | 0.044 | 8.7 |
| Espírito Santo | 0.401 | 0.043 | 9.6 |
| Goiás | 0.534 | 0.060 | 7.3 |
| Maranhão | 0.363 | 0.040 | 7.0 |
| Mato Grosso | 0.323 | 0.038 | 7.7 |
| Mato Grosso do Sul | 0.429 | 0.059 | 11.2 |
| Minas Gerais | 0.357 | 0.035 | 7.9 |
| Pará | 0.362 | 0.046 | 8.5 |
| Paraíba | 0.483 | 0.048 | 8.1 |
| Paraná | 0.269 | 0.029 | 8.6 |
| Pernambuco | 0.421 | 0.044 | 6.0 |
| Piauí | 0.248 | 0.022 | 6.9 |
| Rio de Janeiro | 0.597 | 0.069 | 8.1 |
| Rio Grande do Norte | 0.309 | 0.032 | 7.6 |
| Rio Grande do Sul | 0.657 | 0.066 | 6.1 |
| Rondônia | 0.613 | 0.083 | 15.3 |
| Roraima | 0.362 | 0.051 | 15.5 |
| Santa Catarina | 0.405 | 0.046 | 9.9 |
| São Paulo | 0.373 | 0.047 | 12.4 |
| Sergipe | 0.515 | 0.055 | 11.0 |
| Tocantins | 0.250 | 0.020 | 10.7 |
