# Supplementary material for: Quantifying gaps in the tuberculosis care cascade in Brazil: A mathematical model study using national program data
Source: PLoS Med. 2024 Mar 21;21(3):e1004361. doi: 10.1371/journal.pmed.1004361 (PMC10994550; doi:10.1371/journal.pmed.1004361)
Supplement: S3 Table — (DOCX) [file pmed.1004361.s010.docx]

**Table S7.** Description of modeled health system scenarios and associated parametric adjustments.

| **Scenario** | **Parameter adjustments** |
| --- | --- |
| No delays to diagnosis | Rate of presentation for diagnosis -> 1000x for all states |
| No false negative diagnoses | Sensitivity -> 1 |
| No false positive diagnoses | Specificity -> 1 |
| Rifampin resistance identified at initial diagnosis | Probability of rifampicin resistance test on diagnosis -> 1 |
| No pre-treatment loss to follow up | Primary loss to follow-up fraction -> 0 |
| No treatment loss to follow up | Loss to follow up rate -> 0 |
| No treatment failure | Probability of cure upon treatment completion -> 1 |
| No delay in retreatment after treatment failure | Treatment failure identification probability -> 1 |
| No post-TB sequelae after TB cure | Mortality risk ratio of cured TB -> 1; disability weight of cured TB -> 0 |
| No TB (reference) | Mortality for all states -> background; disability weight associated with TB disease -> 0 |
