# Supplementary material for: Quantifying gaps in the tuberculosis care cascade in Brazil: A mathematical model study using national program data
Source: PLoS Med. 2024 Mar 21;21(3):e1004361. doi: 10.1371/journal.pmed.1004361 (PMC10994550; doi:10.1371/journal.pmed.1004361)
Supplement: S4 Table — Scenario-based burden of disease assessment for subgroups of cases with HIV coinfection (A) and RR-TB (B). Parentheses indicate 95% UI in simulations of parametric uncertainty. TB: tuberculosis. (DOCX) [file pmed.1004361.s011.docx]

**Table S10**. Scenario-based burden of disease assessment for subgroups of cases with HIV coinfection (A) and RR-TB (B). Parentheses indicate 95% UI in simulations of parametric uncertainty.

**A**

| **Scenario** | **Total TB Deaths** | | **Total TB Attributable DALYs** | | **Total TB Programmatic Costs** | |
| --- | --- | --- | --- | --- | --- | --- |
|  | **Value** | **Percent change** | **Value (thousands)** | **Percent change** | **Value (millions USD)** | **Percent change** |
| ***Reference scenario*** | | | | | | |
| Current programmatic performance | 1880  (1420, 2490) | ----- | 32.8  (26.4, 38) | ----- | 8.0  (6.3, 10.0) | ----- |
| ***Alternative scenarios*** | | | | | | |
| No delays to diagnosis | 620  (450, 840) | -67.3  (-72.1, -61.1) | 8.9  (6.7, 11.4) | -72.9  (-77.5, -67.8) | 9.0  (7.4, 11.0) | 15.6  (8.6, 26.3) |
| No false negative diagnoses | 1830  (1390, 2440) | -2.9  (-4.8, -1.4) | 32.0  (25.7, 37.0) | -2.5  (-4.0, -1.2) | 7.8  (6.4, 9.7) | -0.4  (-1.3, 0.4) |
| Rifampin resistance identified at initial diagnosis | 1870  (1420, 2480) | -0.4  (-0.7, -0.2) | 32.7  (26.3, 37.9) | -0.4  (-0.6, -0.1) | 7.8  (6.4, 9.7) | 0.1  (-0.6, 1.1) |
| No pre-treatment loss to follow up | 1860  (1410, 2460) | -1.1  (-1.4, -0.8) | 32.5  (26.2, 37.6) | -0.9  (-1.2, -0.7) | 7.8  (6.4, 9.7) | -0.1  (-0.4, 0.1) |
| No treatment loss to follow up | 1720  (1280, 2350) | -8.4  (-12.0, -5.3) | 30.5  (24.7, 35.7) | -7.0  (-10.1, -4.5) | 7.9  (6.5, 9.7) | 1.3  (-0.7, 3.8) |
| No treatment failure | 1820  (1390, 2410) | -3.2  (-6.5, -1.2) | 32  (25.3, 37.3) | -2.6  (-4.7, -1.0) | 7.6  (6.2, 9.5) | -2.3  (-4.3, -1.0) |
| No delays in retreatment after treatment failure | 1850  (1400, 2460) | -1.4  (-2.8, -0.7) | 32.4  (26.0, 37.7) | -1.2  (-2.1, -0.6) | 7.9  (6.5, 9.8) | 1.8  (0.9, 2.8) |
| No post-TB sequelae after TB cure | 1880  (1420, 2490) | ----- | 26.1  (20.3, 32.0) | -20.7  (-27.7, -15.1) | 7.8  (6.4, 9.7) | ----- |

**B**

| **Scenario** | **Total TB Deaths** | | **Total TB Attributable DALYs** | | **Total TB Progammatic Costs** | |
| --- | --- | --- | --- | --- | --- | --- |
|  | **Value** | **Percent change** | **Value (thousands)** | **Percent change** | **Value (millions USD)** | **Percent change** |
| ***Reference scenario*** | | | | | | |
| Current programmatic performance | 300  (190, 420) | ----- | 8.6  (5.5, 11.8) | ----- | 12.2  (8.0, 16.4) | ----- |
| ***Alternative scenarios*** | | | | | | |
| No delays to diagnosis | 230  (150, 330) | -22.3  (-28.7, -16.9) | 6.4  (4.0, 8.9) | -26  (-32.9, -19.8) | 13.6  (9.1, 18.1) | 11.8  (8.5, 15.0) |
| No false negative diagnoses | 300  (180, 410) | -1.2  (-2.0, -0.6) | 8.5  (5.5, 11.6) | -1.3  (-2.1, -0.6) | 12.2  (8.0, 16.4) | 0.6  (0.3, 1.0) |
| Rifampicin resistance identified at initial diagnosis | 240  (150, 330) | -20.2  (-28.4, -10.1) | 7.0  (4.5, 9.4) | -18.6  (-26.1, -9.6) | 11.6  (7.6, 15.8) | -4.2  (-12.2, 6.2) |
| No pre-treatment loss to follow up | 300  (190, 420) | -0.4  (-0.6, -0.3) | 8.5  (5.5, 11.7) | -0.4  (-0.6, -0.3) | 12.2  (8.0, 16.4) | 0.2  (0.1, 0.3) |
| No treatment loss to follow up | 270  (170, 380) | -9.6  (-12.5, -6.9) | 7.8  (5.0, 10.5) | -9.2  (-12.4, -6.7) | 12.6  (8.3, 17.1) | 3.8  (1.5, 6.2) |
| No treatment failure | 290  (180, 400) | -4.2  (-6.3, -2.6) | 8.3  (5.3, 11.3) | -3.7  (-5.5, -2.3) | 11.6  (7.7, 15.6) | -4.6  (-6.9, -2.8) |
| No delay in retreatment after treatment failure | 260  (160, 360) | -13.2  (-17.7, -10.1) | 7.5  (4.8, 10.2) | -13  (-17.7, -9.7) | 13.3  (8.8, 18.0) | 9.7  (5.6, 14.7) |
| No post-TB sequelae after TB cure | 300  (190, 420) | ----- | 6.9  (4.3, 9.8) | -19.4  (-25.6, -14.9) | 12.2  (8.0, 16.4) | ----- |
