# Supplementary material for: Quantifying gaps in the tuberculosis care cascade in Brazil: A mathematical model study using national program data
Source: PLoS Med. 2024 Mar 21;21(3):e1004361. doi: 10.1371/journal.pmed.1004361 (PMC10994550; doi:10.1371/journal.pmed.1004361)
Supplement: S1 TRIPOD Checklist — (PDF) [file pmed.1004361.s012.pdf]

TRIPOD Checklist: Prediction Model Development

Section/Topic	Item	Checklist Item	Page
**Title and abstract**			
Title	1	Identify the study as developing and/or validating a multivariable prediction model, the target population, and the outcome to be predicted.	**Title**
Abstract	2	Provide a summary of objectives, study design, setting, participants, sample size, predictors, outcome, statistical analysis, results, and conclusions.	**Abstract**
**Introduction**			
Background and objectives	3a	Explain the medical context (including whether diagnostic or prognostic) and rationale for developing or validating the multivariable prediction model, including references to existing models.	**Introduction P1-3**
	3b	Specify the objectives, including whether the study describes the development or validation of the model or both.	**Introduction P4**
**Methods**			
Source of data	4a	Describe the study design or source of data (e.g., randomized trial, cohort, or registry data), separately for the development and validation data sets, if applicable.	**Methods P2**
	4b	Specify the key study dates, including start of accrual; end of accrual; and, if applicable, end of follow-up.	**Methods P2**
Participants	5a	Specify key elements of the study setting (e.g., primary care, secondary care, general population) including number and location of centres.	**Methods P1-2**
	5b	Describe eligibility criteria for participants.	**Methods P1**
	5c	Give details of treatments received, if relevant.	**NA**
Outcome	6a	Clearly define the outcome that is predicted by the prediction model, including how and when assessed.	**Methods P8-11**
	6b	Report any actions to blind assessment of the outcome to be predicted.	**NA**
Predictors	7a	Clearly define all predictors used in developing or validating the multivariable prediction model, including how and when they were measured.	**Methods P3-7**
	7b	Report any actions to blind assessment of predictors for the outcome and other predictors.	**NA**
Sample size	8	Explain how the study size was arrived at.	**NA**
Missing data	9	Describe how missing data were handled (e.g., complete-case analysis, single imputation, multiple imputation) with details of any imputation method.	**Methods P3-7**
Statistical analysis methods	10a	Describe how predictors were handled in the analyses.	**Methods P3-7**
	10b	Specify type of model, all model-building procedures (including any predictor selection), and method for internal validation.	**Methods P3-7**
	10d	Specify all measures used to assess model performance and, if relevant, to compare multiple models.	**Methods P8-10**
Risk groups	11	Provide details on how risk groups were created, if done.	**Methods P5**
**Results**			
Participants	13a	Describe the flow of participants through the study, including the number of participants with and without the outcome and, if applicable, a summary of the follow-up time. A diagram may be helpful.	**NA**
	13b	Describe the characteristics of the participants (basic demographics, clinical features, available predictors), including the number of participants with missing data for predictors and outcome.	**NA**
Model development	14a	Specify the number of participants and outcome events in each analysis.	**Results P1**
	14b	If done, report the unadjusted association between each candidate predictor and outcome.	**NA**
Model specification	15a	Present the full prediction model to allow predictions for individuals (i.e., all regression coefficients, and model intercept or baseline survival at a given time point).	**NA**
	15b	Explain how to the use the prediction model.	**NA**
Model performance	16	Report performance measures (with CIs) for the prediction model.	**Methods P8-10**
**Discussion**			
Limitations	18	Discuss any limitations of the study (such as nonrepresentative sample, few events per predictor, missing data).	**Discussion P7**
Interpretation	19b	Give an overall interpretation of the results, considering objectives, limitations, and results from similar studies, and other relevant evidence.	**Discussion P1**
Implications	20	Discuss the potential clinical use of the model and implications for future research.	**Discussion P8**
**Other information**			
Supplementary information	21	Provide information about the availability of supplementary resources, such as study protocol, Web calculator, and data sets.	**Figure/Table S1-S11**
Funding	22	Give the source of funding and the role of the funders for the present study.	**included**

We recommend using the TRIPOD Checklist in conjunction with the TRIPOD Explanation and Elaboration document.
